# Midline Prostatic Cyst Marsupialization Using Holmium Laser

**DOI:** 10.1155/2015/797061

**Published:** 2015-05-26

**Authors:** Mehmet Kilinc, Yunus Emre Goger, Mesut Piskin, Mehmet Balasar, Abdulkadir Kandemir

**Affiliations:** ^1^Urology Department, Meram Medical Faculty, Necmettin Erbakan University, 42080 Konya, Turkey; ^2^Urology Department, Konya Education and Research Hospital, 42090 Konya, Turkey

## Abstract

Many of the prostatic cysts are asymptomatic and only 5% are symptomatic (Hamper et al., 1990; Higashi et al., 1990). These symptoms include pelvic pain, hematospermia, infertility, voiding dysfunction, prostatitis-like syndrome, and painful ejaculation. Treatment of prostatic cysts includes TRUSG guided drainage, endoscopic transurethral resection, and in some cases even open surgery. In the literature, endoscopic interventions use marsupialization of the midline prostatic cyst with transurethral resection (TUR) or transurethral incision with endoscopic urethrotomy (Dik et al., 1996; Terris, 1995). Holmium: YAG laser was employed for the marsupialization of the cyst wall in midline prostatic cyst treatment for the first time in the present study. Symptoms, treatment, and follow-up are presented in this paper.

## 1. Introduction

Midline prostatic cyst presence in the male population is extremely rare with an incidence of less than 1% [1]. However, recent imaging techniques have made cysts incidences more prevalent (5% to 8.6%) [2, 3].

Prostatic cysts can be categorized as Mullerian duct cysts, utriculus prostatic cysts, ejaculatory duct cysts, seminal vesicle cysts, and prostatic retention cysts [4]. These categorizations are based on cyst location, shape, embryogenic origin, interconnection with prostatic urethra or seminal vesicle, and sperm presence in the cyst.

Midline prostatic cysts are frequently diagnosed incidentally or based on symptoms in the lower urinary tract. These might include urinary obstruction, painful ejaculation, hematospermia, urinary tract infections, infertility, chronic pelvic pain, and prostatitis-like syndrome symptoms [5, 6].

Midline prostatic cysts treatment modalities include transrectal or perineal ultrasound-guided drainage, endoscopic transurethral resection, and open surgery.

In the literature, endoscopic interventions use marsupialization of the midline prostatic cyst with transurethral resection (TUR) or transurethral incision with endoscopic urethrotomy [6, 7]. Holmium: YAG laser was employed for the marsupialization of the cyst wall in midline prostatic cyst treatment for the first time in the present study. Symptoms, treatment, and follow-up are presented below.

## 2. Case Report

A 26-year-old male patient was admitted to our clinic with a history of chronic pelvic pain history of more than 2 years, occasional genital discharge, frequent urinary tract infection, dysuria, and painful ejaculation.

**Figure 1:**
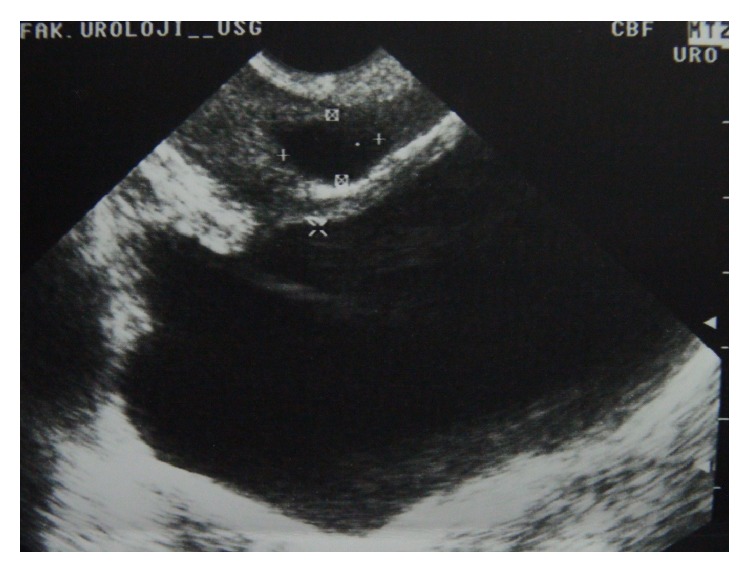
Prostatic midline cyst TRUSG.

**Figure 2:**
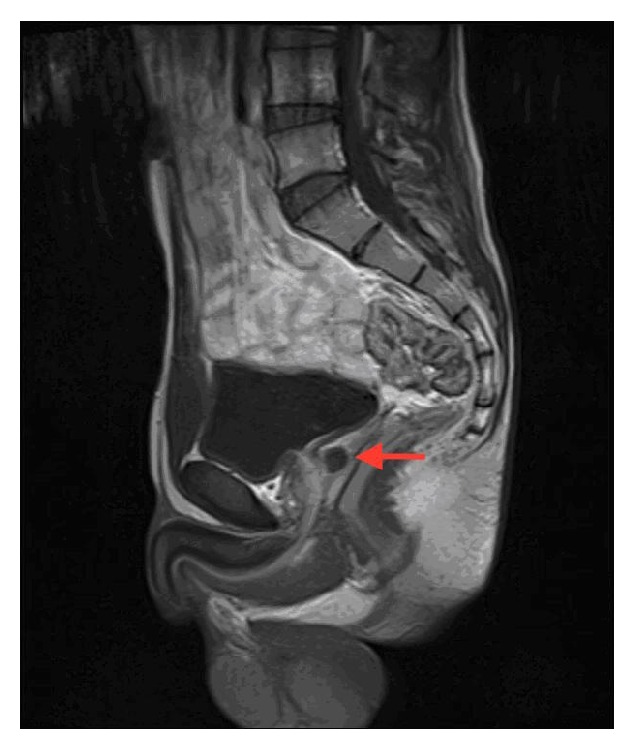
Prostatic midline cyst MRI.

Genital examination, external meatus, and digital rectal examination of the patient revealed normal findings. Patient's IPSS was 10 without pathology in urine examination or culture. Transrectal ultrasound (TRUSG) revealed a midline prostatic cyst with approximately 10 × 12 mm diameter (Figure 1). Prostatic cyst and urethra distance was 5 mm. No abnormality was seen in the seminal vesicles. The pelvic MRI verified the same midline prostatic cyst of 12 × 10 mm diameter (Figure 2). Minimally invasive endoscopic intervention using holmium: YAG laser was considered to treat long-term lower urinary system symptoms in order to minimize potential harm inflicted on the urogenital tract and future sexuality of the young patient.

**Figure 3:**
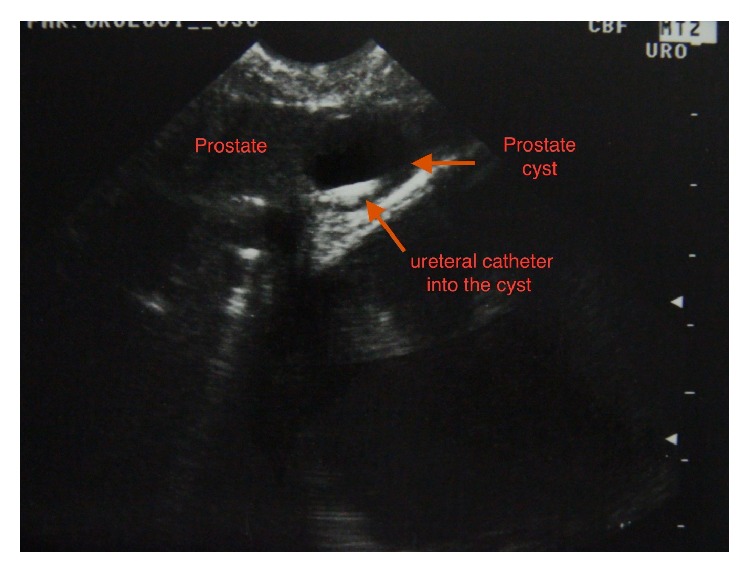
Ureter catheter in prostatic cyst.

**Figure 4:**
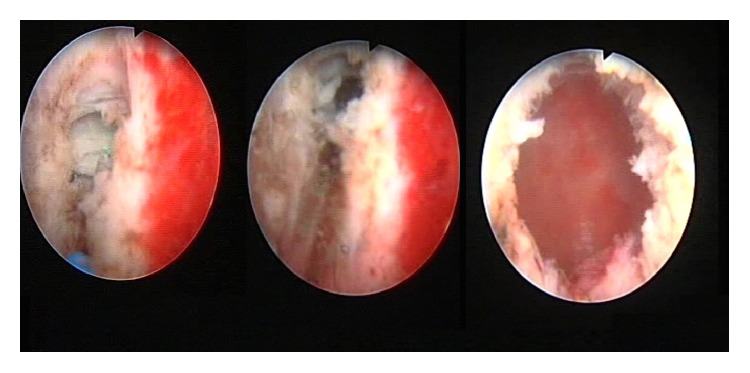
Prostate cyst marsupialization with holmium: YAG laser.

Under general anesthesia, a 16 Fr cystoscope was inserted into the urethra. 3 Fr ureteral catheter was inserted through the opening of the utriculus as there was no urethral stricture. Simultaneously, TRUSG performed showed the 3 Fr ureteral catheter in the cysts (Figure 3). Following aspiration, the specimen obtained was referred to microbial culture and pathology analyses. Afterwards, beginning at the cyst's opening, marsupialization of the cyst wall using the holmium: YAG laser was performed with 12-watt energy (Dornier Medilas Holmium Laser H 20) under ureteral catheter guidance (Figure 4). Subsequently, a 16 Fr Foley catheter was inserted at the end of the intervention. Holmium laser usage enabled easier and more controlled tissue excision and tissue bleeding. On the 1st postoperative day, the urethral catheter was removed and the patient was discharged. The pathology report of the aspirated content described the existence of malignant cells only.

## 3. Discussion

Prostatic cysts are mostly asymptomatic. Only 5% prostatic cysts are symptomatic. Associated symptoms include pelvic pain, hematospermia, infertility, voiding dysfunction, prostatitis-like syndrome, and painful ejaculation (5.6). Midline prostatic cysts are generally Mullerian duct or utriculus cysts [8]. Midline cysts, located posteriorly at the prostatic floor, are mostly of developmental origin and arise from remnants of fetal tissue—in the utricle or Mullerian duct. Whereas Mullerian cysts are mesodermal in origin, contain spermatozoa, and are located more posteriorly nearer to the prostate base, utricular cysts are mostly endodermal, contain no spermatozoa, and are located near the verumontanum [8].

The necessary tests for a patient with a midline prostatic cyst include medical history, urinalysis, TRUSG, and pelvic MRI [9–11]. Treatment of prostatic cysts in the literature includes TRUSG guided drainage, endoscopic transurethral resection, and in some cases even open surgery. Endoscopic methods include either transurethral resection or incision of the cyst with an endoscopic urethrotomy knife. In the present case, an endoscopic intervention was chosen for the presence of frequent urinary tract infections, painful ejaculation resistant to medical treatment, and prostatitis syndrome-like symptoms. The distance between the cyst and the urethra was measured using TRUSG prior to endoscopic intervention. This enabled us to determine cyst resection degree over the verumontanum [12]. Using the ureteral catheter as a guide, beginning from the cyst's opening, marsupialization of the cyst wall was conducted with holmium: YAG laser. In midprostatic cysts, endoscopic complications after TUR are rare (0.5%–2.4%) and in the form of urethral stricture and bladder neck stenosis [13]. A similar endoscopic intervention study conducted with 17 patients reported urethral stenosis in one case and bladder neck stenosis in another [14]. However, other studies with 11 and 18 patients were totally complication-free [6, 15]. Although complications are rare, the prevalence of midline prostatic cysts in younger patients may frequently lead to more problematic complications. In order to minimize the risk of complications, cystoscopy was performed using a 16 Fr cystoscope. The process was completed using the holmium laser with minimal trauma to the bladder neck and urethra. Holmium laser usage enabled easier and more controlled tissue excision and tissue bleeding. During the 3- and 6-month follow-up periods, no complications were observed. Pelvic pain and painful ejaculation disappeared.

## 4. Conclusion

In patients with urologic symptoms detection of midline cysts will require a focused examination as they can represent a normal cyst variation or actually be the underlying cause. In symptomatic patients with midline prostatic cyst, holmium laser technology, as a minimally invasive method enabling the use of lower-Fr endoscopic instrument, is expected to become more prominent. However, further studies with larger patient series are required before holmium: YAG laser use becomes the standard treatment modality in symptomatic midline prostatic cyst treatment.
